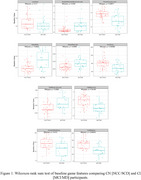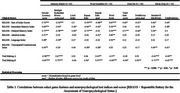# Short‐interval common game play distinguishes people with and without cognitive impairment

**DOI:** 10.1002/alz70856_103733

**Published:** 2025-12-25

**Authors:** Jake A Galler, Liuqing Yang, Chao‐Yi Wu, Cathrine Young, Edmarie Guzman‐Velez, Anthony W. Bannon, Hiroko H Dodge, Jessica A. Gerber, Steven E Arnold

**Affiliations:** ^1^ Massachusetts General Hospital, Harvard Medical School, Boston, MA, USA; ^2^ AbbVie Inc., North Chicago, IL, USA

## Abstract

**Background:**

Many efforts have been made to adapt neuropsychological assessments to digital platforms for easier and more frequent measurement of cognitive functioning. However, these still require a participant's concerted effort and can be tiresome for the cognitively impaired. Moreover, compliance within in‐home settings is often poor, and the reliability of measurements is uncertain. To address these limitations, we took a converse approach by “cognifying” familiar and enjoyable games for tablet play to capture neurocognitive data. The current study evaluates the MIND GamePack^©^'s effectiveness in differentiating cognitive status through two weeks of gameplay, with further validation against standard neuropsychological tests.

**Method:**

The MIND GamePack^©^ includes four iPadOS games: Memory Match (pair matching), Word Scramble (Boggle), FreeCell (Solitaire), and Block Drop (Tetris). Sixty participants were assessed: 37 cognitively normal (CN) with no cognitive complaints (NCC, *n* = 26), or with subjective cognitive complaints (SCD, *n* = 11), and 23 cognitively impaired (CI) with either mild cognitive impairment (MCI, *n* = 15) or mild dementia (MD, *n* = 8). Participants were instructed to play each game for a minimum of five minutes/day, five days/week. Ten gameplay features summarized from two weeks of gameplay were analyzed across cohorts using Wilcoxon rank sum test. Spearman's correlation was used to validate digital biomarkers against RBANS indices and Trail Making Test (TMT).

**Result:**

Nine of ten features differentiated normal cognition [NCC/SCD] from impairment [MCI/MD], with CI participants performing worse across features [Figure 1] *Percent Accuracy* displays a high correlation with RBANs Immediate & Delayed Memory Indices [*r* = 0.60 to 0.75]. Similarly, *Number of Hard Drops* exhibits a strong association with TMT A & B [*r* = ‐0.61 to ‐0.71], while *Word Score* demonstrates a moderate association with RBANs Language Index [*r* = 0.5] [Table 1.]

**Conclusion:**

Features of the MIND GamePack^©^ effectively differentiate CN from CI individuals over a short period with digital biomarkers highlighting memory, language, and executive function differences. Gamified assessments can be incorporated into clinical trials to facilitate screening, track performance over time, and may serve as sensitive measures of cognitive stability, decline, or response.